# Navigating the Decision to Discontinue Intravitreal Injection Therapy in End-Stage Neovascular Age-Related Macular Degeneration

**DOI:** 10.3390/jpm15100487

**Published:** 2025-10-13

**Authors:** Justin Bennie, David J. Ramsey

**Affiliations:** 1 Wayne State University School of Medicine, 540 E Canfield St, Detroit, MI 48201, USA; gh9886@wayne.edu; 2 Division of Ophthalmology, Department of Surgery, UMass Chan—Lahey School of Medicine, 31 Burlington Mall Rd, Burlington, MA 01805, USA; 3 Department of Ophthalmology, Tufts University School of Medicine, Boston, MA 02111, USA; 4 Biomedical Sciences and Disease, New England College of Optometry, 424 Beacon St, Boston, MA 02115, USA

**Keywords:** age-related macular degeneration (AMD), intravitreal injections, vascular endothelial growth factor (VEGF), low vision, quality of life

## Abstract

**Introduction:** The management of neovascular age-related macular degeneration (nAMD) is constrained by diminishing therapeutic options for retina specialists and their patients when the disease reaches its end stages. **Methods:** Clinical insights emerge from two case narratives in which patients benefitted from discontinuation of anti-VEGF therapy. **Results:** Long-term management of nAMD with intravitreal injections of agents targeting vascular endothelial growth factor (VEGF) is crucial for slowing progression of the disease and is generally well-tolerated. However, vision often declines as the disease progresses over time, even with treatment. This article presents strategies for aligning therapeutic goals with their expected visual outcome when an eye has reached end-stage disease. It addresses considerations for how and when to stop treatment when vision becomes limited, taking into consideration the visual status of the fellow eye and incorporating input from low vision specialists who can better assess best-corrected visual acuity (BCVA) and optimize the visual function of patients. We also acknowledge the potential benefits of switching either the dose or the agent that targets VEGF to alter the long-term visual outcome of treatment. Finally, we discuss the importance of taking into consideration related manifestations of the disease, such as macular scarring, geographic atrophy, or other retinal or optic nerve diseases which may limit vision and thus the utility of continued nAMD treatment. **Conclusions:** Building a strong patient–physician relationship is essential for navigating the shared decision-making process of when to stop treatment for nAMD.

## 1. Introduction

Age-related macular degeneration (AMD) is a leading cause of visual impairment and blindness among adults aged 50 and older and affects more than 200 million people worldwide [1]. As the population ages and life expectancy improves, the prevalence of advanced AMD will also increase—presently, more than 200,000 new cases of neovascular age-related macular degeneration (nAMD) are diagnosed each year in North America [2].

Agents targeting vascular endothelial growth factor (VEGF) have revolutionized the management of nAMD. Treatment of nAMD most often requires regular intravitreal injections of anti-VEGF medications, initially given monthly, then often followed by extended intervals between treatments [3]. While intervention is usually highly effective at preventing the rapid progression of the disease by controlling macular neovascularization and associated exudation or hemorrhage and modestly improving short-term vision, the disease typically progresses, causing declines in vision despite treatment [4]. Many patients develop scarring in the macula, even as macular neovascularization or hemorrhage regresses with treatment. One study reported that nearly half of patients being treated for nAMD developed fibrotic and nonfibrotic changes within two years of starting treatment [5]. Still other patients go on to develop geographic atrophy, a late stage of advanced dry AMD, the onset of which may be hastened in eyes treated for nAMD [6,7]. Together, these conditions account for significant, irreversible vision loss.

In this article, we address the considerations that should ideally inform the decision of when to stop treatment with intravitreal injections of agents targeting VEGF after an eye has reached the end stages of the disease. We also review previously published data that help inform aligning the goals of therapy with their expected visual outcomes. While classifying the disease as “end stage” may include a variety of underlying pathologies and factors, the findings of vision-limiting geographic atrophy or fibrosis are the most significant features of such advanced-stage disease. Whereas geographic atrophy is a chronic, progressive degeneration of the macula [6,7], fibrosis is a physiological response that aims to restore anatomical integrity when organ tissue may otherwise cease to function or function minimally [8]. In this report, we highlight the experience of two patients with late-stage nAMD, each of whom made the decision with their retina provider to stop intravitreal injections after concluding that a sufficient treatment trial had failed to deliver any meaningful improvement in an eye with limited vision. While maintenance of nAMD with intravitreal anti-VEGF therapy has been proven to be safe and effective at preserving vision even when used at extended intervals [9], some eyes fail to maintain visual function or reach advanced stages where vision is unlikely to deteriorate even when treatment is withheld [10]. In such cases, it is desirable to engage patients in a frank discussion of the goals of therapy and at what point intravitreal injections are best discontinued, in view of the patient’s experience of the disease, the risks associated with intravitreal injection therapy, and the economic and social costs resulting from treatment. Regrettably, the decision process surrounding stopping injection therapy for individuals with end-stage nAMD is rarely formally addressed and lacks formal guidelines.

## 2. Materials and Methods

The study was conducted in accordance with the Declaration of Helsinki and approved by the Institutional Review Board of the Lahey Hospital & Medical Center in Burlington, MA, USA (Protocol #2017-007, approved 23 December 2016) and deemed exempt from further review by virtue of being a report of two cases. Information in this study report was gathered and secured in compliance with the Health Insurance Portability and Accountability Act. The two patients featured in this study provided written informed consent for inclusion. This case report was prepared in accordance with the CARE (CAse REport) guidelines (https://www.care-statement.org). A thorough review of the demographic, clinical, imaging, and social features of each case was conducted. Best-corrected visual acuity (BCVA) was measured using the Snellen chart. Acquisition of spectral-domain OCT of the macula was acquired with a SPECTRALIS HRA+OCT device (Heidelberg Engineering GmbH, Heidelberg, Germany). Cases were selected based on patient availability and a willingness to share unique personal experiences. Patient-reported outcome measures were based on interviews conducted after discontinuing anti-VEGF therapy.

## 3. Results

### 3.1. Case 1

**Figure 1:**
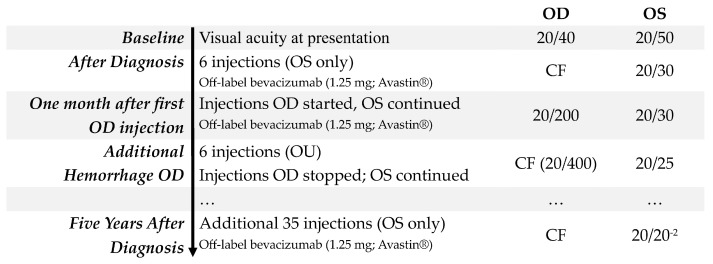
Timeline illustrating diagnosis and progression of treatment of nAMD in Case 1, a woman who developed nAMD at age 89, initially in the right eye and subsequently in the left eye.

**Figure 2:**
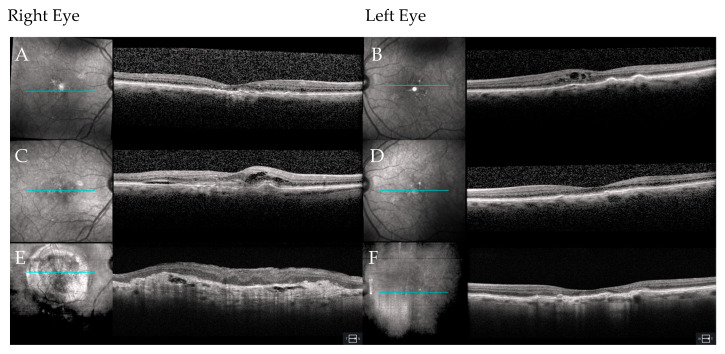
OCT imaging of the right (**A**) and left (**B**) eyes at diagnosis of nAMD in the left eye in Case 1 (age 89). The right eye demonstrates RPE disruption with subretinal drusen deposits, while the left eye illustrates intraretinal and subretinal fluid with exudates. OCT imaging at the visit when treatment was discontinued in the right eye (**C**). Subretinal hyperreflective material consistent with subretinal hemorrhage seen clinically is evident along with significant subretinal and intraretinal fluid. By contrast, the left eye (**D**) is now free of any activity from the nAMD. Finally, OCT imaging of right eye at last follow-up (age 94) illustrates a disciform scar with hyperreflective material and geographic atrophy (**E**), while the left eye (**F**) remains free of nAMD activity, but now shows non-subfoveal geographic atrophy. In each panel, a B-scan locator line (blue) on the infrared reflectance fundus image marks the position of the corresponding OCT cross-section.

An 89-year-old woman was originally referred to the retina service for routine monitoring of intermediate AMD by an optometrist after she was diagnosed with the disease three years earlier. At her scheduled follow-up visit, she admitted to a three-to-four-month history of mild visual distortion in her left eye when checking her home Amsler grid. This immediately raised concern for nAMD. Upon examination, her visual acuity was 20/40 in the right eye and 20/50 in the left eye (Figure 1). This represented a modest decline of one line below her prior level of vision in the left eye evaluated six months earlier. Anterior segment findings were normal, and she was bilaterally pseudophakic. Examination of the fundus of the right eye identified a large- and intermediate-sized drusen with associated retinal pigment epithelial (RPE) changes. The fellow left eye had an even greater quantity of drusen and had macular thickening, suggesting a neovascular membrane. Optical coherence tomography (OCT) imaging demonstrated stable drusen with a pigment epithelial detachment in the right eye. In contrast, the left eye identified new intra- and subretinal fluid with a small amount of subretinal hyperreflective material (Figure 2A,B). The patient was informed that these findings likely represented nAMD. She declined fluorescein angiography in favor of empiric treatment with off-label bevacizumab (1.25 mg; Avastin^®^; Genentech Inc., San Francisco, CA, USA).

The patient completed a series of three-monthly injections of bevacizumab at four-week intervals. After responding well to this initial treatment, she elected to follow a treat-and-extend protocol to reduce the frequency of visits and intravitreal injections. She received six injections in the left eye over the next eight months. The following summer her vision in the left eye had improved to 20/30, but her right eye suddenly declined to count fingers (CF), prompting an early return visit. Clinical examination was notable for a new subretinal hemorrhage in the macula of the right eye with associated exudation. OCT imaging of the macula identified subretinal hyperreflective material, as well as intra- and subretinal fluid (Figure 2C,D). A trial of three-monthly injections of off-label bevacizumab (1.25 mg) was started in the right eye, after which her vision improved to 20/200 but later declined to 20/400 and then to CF when she suffered another hemorrhage five months after the first. During this period, she expressed frustration over the lack of improvement her right eye but remained on injections of bevacizumab every five to six weeks in her left eye, which remained stable with BCVA of 20/25. She was referred to a low vision specialist. Despite a careful low vision refraction, the patient was only able to achieve a BCVA of 20/400. In total, she received six intravitreal injections in both her right and left eyes over this eight-month period before concluding that treatment of her right eye was not contributing meaningfully to her vision-associated quality of life. Treatment was therefore stopped, as it was concluded that she had reached end-stage disease with a disciform scar with CF vision in the right eye. At this visit macular OCT imaging revealed progressive macular scarring with associated geographic atrophy. Subsequent to that visit, she continued to receive injections in her left eye at eight-to-ten-week intervals. Now five years later, at age 94, her left eye BCVA reached 20/20^−2^ while her right eye remained CF (Figure 2E,F).

The patient continues to live on her own but relies on both her son and her daughter to take her to her eye appointments. When asked about her experience with treatment for her nAMD, past and present, she reported that while she found eye injections “uncomfortable, particularly when given in both eyes on the same day,” she was disappointed when they were stopped in the one eye because she had hoped they would continue to improve her vision. She credits her trust in her physician with providing her the confidence to come to terms with stopping injections in her right eye. She describes this shared patient–physician decision to continue treatment in one eye instead of both as “difficult at the time, but I reached the decision because the good [near 20/20] vision in my remaining eye,” which continued vision-preserving treatment over nearly a half-decade since its diagnosis. At no point did the patient request to resume treatment in her right eye, noting that it “has such limited vision”.

### 3.2. Case 2

**Figure 3:**
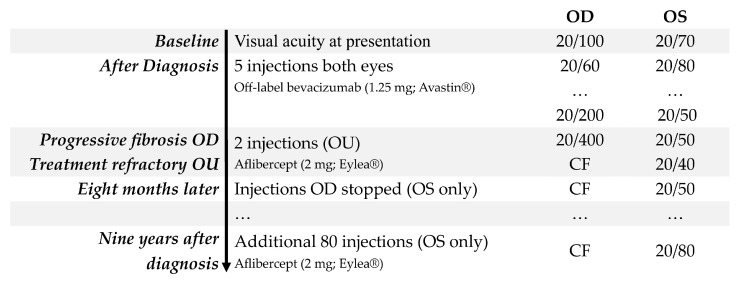
Timeline illustrating the diagnosis and progression of treatment of nAMD in Case 2, a woman who developed bilateral nAMD at age 80.

**Figure 4:**
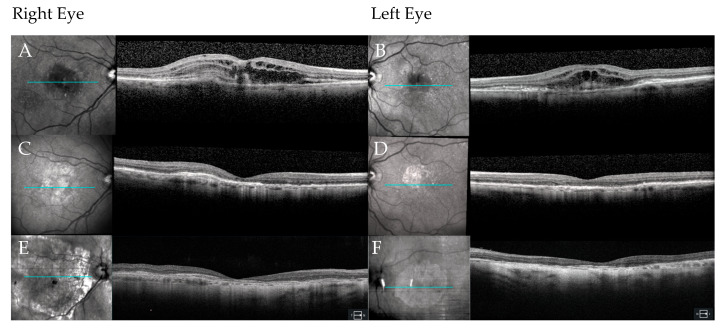
OCT imaging of the right (**A**) and left (**B**) eyes at diagnosis in Case 2 (age 80) show bilateral exudation with subretinal hyperreflective material consistent with subretinal hemorrhage. OCT imaging at the visit when treatment was discontinued in the right eye (**C**) demonstrates subretinal hyperreflective material consistent with subretinal fibrosis. By contrast, the left eye (**D**) is now free of any activity from the nAMD. Finally, OCT imaging of right eye at last follow-up (age 89) illustrates a disciform scar with geographic atrophy (**E**), while the left eye (**F**) remains free of activity from nAMD activity, but also shows non-subfoveal geographic atrophy. In each panel, a B-scan locator line (blue) on the infrared reflectance fundus image marks the position of the corresponding OCT cross-section.

An 80-year-old woman presented with a complaint of decreased vision in both eyes accompanied by a dull headache. Despite obtaining new glasses two months earlier, at which time her vision was 20/40 OU with the limitation in BCVA attributed to intermediate AMD in both eyes, she noted that her vision had not improved and had, in fact, painlessly worsened over the subsequent few months. She made a routine appointment to see an optometrist who diagnosed bilateral nAMD and made a same-day referral to the retina clinic. Upon examination, her BCVA was 20/100 in the right eye and 20/70 in the left eye (Figure 3). This represented a substantial decline from her prior level of BCVA in both eyes. Anterior segment findings were normal, and she was bilaterally pseudophakic. Clinical examination showed subretinal hemorrhage in both eyes with signs of early fibrosis, indicating a chronic exudation in both eyes. OCT imaging of the macula confirmed the diagnosis of nAMD with hyperreflective changes consistent with choroidal neovascular membrane and inner- and subretinal fluid bilaterally (Figure 4A,B).

A trial of three-monthly injections of off-label bevacizumab (1.25 mg) was started in both eyes. Visual and anatomical improvement was noted in both eyes with her vision improving to 20/60 in the right eye and eventually 20/50 in the left eye. Whereas the visual acuity in the left eye continued to improve, the right eye worsened to 20/200 by the fifth injection of off-label bevacizumab. Both eyes were deemed treatment-refractory at this point, each with a small amount of intraretinal and subretinal fluid. The patient was informed that the right eye had a guarded prognosis because of increasing subretinal fibrosis, observed as hyperreflective material located between the posterior boundary of the neuroretina and the RPE on OCT imaging [11]. The decision was made to switch to intravitreal aflibercept (2 mg; Eylea^®^, Regeneron Pharmaceuticals, Inc., Tarrytown, NY, USA). Both eyes responded to this new medication with a reduction in exudation, but only the left eye showed improvement in vision, improving to 20/40. She was sent to a low vision specialist at this time; however, her BCVA did not improve in either eye. With the right eye remaining at CF and after several discussions about treatment goals, the decision was made to stop treatment after two of the three-monthly loading doses of aflibercept (2 mg) (Figure 4C,D). The left eye, continuing aflibercept (2 mg), achieved a peak vision of 20/30 a few months later but settled at 20/50 with ongoing monthly injections. Over the subsequent nine years, the patient received an additional 80 aflibercept (2 mg) injections administered at intervals of five to six weeks. The result was good control of fluid in the left eye, but with a gradual decline in BCVA to 20/80 OS. The right eye, by contrast, remained CF without any subjective decline in visual function over this period (Figure 2E,F).

The patient has known her retina specialist for nearly a decade; she lives in a retirement home located a few miles from the clinic, and she uses the transportation provided by the facility to travel to her appointments. During this patient’s interview, she stated that when she and her physician decided to discontinue treatment in the right eye, she was sad but accepting. She credited her trust in her physician as the chief factor in helping her to decide that stopping the injections was the right thing to do. She stated, “I was glad that my physician did not pull punches.” She also expressed gratitude for the realistic treatment goals that had been set in consultation with her physician. When asked about what made her treatment important to her, the patient said, “I value seeing the beautiful earth and so love being able to see my children’s faces and watch them grow.” Looking back at that decision now, she reflects that she is glad that she stopped the injections in the right eye, given the treatment burden and the limited expected benefits. No further deterioration in vision or perceived quality of life occurred after stopping treatment in the right eye, and at no point did the patient elect to resume treatment with bilateral intravitreal anti-VEGF injections after therapy was stopped in the right eye because of poor vision.

## 4. Discussion

The patient–physician relationship is central to the care of patients with nAMD receiving intravitreal injection therapy. This is not only because of the trust required to receive each individual treatment, which is typically given while the patient is awake under local anesthesia, but also because of the ongoing nature of the care required for this chronic, age-related condition [3]. Available clinical evidence also suggests that less-than-optimal visual outcomes result when patients are under-treated relative to receiving monthly injections used in clinical trial protocols [9,12,13]. Though more recent studies of new agents have demonstrated non-inferiority in visual outcomes at longer intervals [14,15]. Unfortunately, many patients inevitably reach the end stages of the disease, despite regular treatment with intravitreal injections of anti-VEGF medications, which are the most effective means for preserving vision [3]. In clinical practice, patients with advanced age also often have a greater number of comorbid illnesses and often present at significantly more advanced stages of disease [16], a feature illustrated by the extent of activity in the two cases presented. Although some eyes can be weaned off treatment [17], most patients require ongoing treatment to control nAMD. Patients may also suffer irreversible vision loss from other aspects of nAMD, for example, regression of choroidal neovascularization is sometimes associated with atrophy of the overlying retina [18].

Both patients described in this report expressed concern about the ramifications of stopping treatment in one of their eyes. In both cases, the eye in question had reached an advanced stage of disease and had impaired visual acuity from hemorrhage and fibrovascular scarring that caused irreversible loss of function of the neurosensory retina [19]. The decision to stop treatment was made easier thanks to the relationship that each patient had with her treating retinal specialist. Ongoing conversations helped each patient understand the goals of treatment and its limitations, and these consultations prepared these individuals for the time when therapy should be discontinued. The involvement of a low vision specialist also provided a degree of reassurance that everything possible to improve vision in the eye had been considered. Unfortunately, many patients with nAMD, including those with low vision, are not referred for such evaluations [20]. It may also be helpful to seek the opinion of another retina provider or ophthalmologist, especially in cases where such consultation can provide additional psychosocial support to a patient faced with having to come to terms with a state of irreversible vision loss. The fact that in both cases presented, each patient had a fellow eye with better vision and was already on intravitreal injection treatment for nAMD likely made the decision to stop treatment in the first eye easier compared with a situation where a fellow eye might have had poor vision. It is also our experience that the decision to stop treatment is more easily accepted when only one eye requires treatment and is not the patient’s better-seeing eye. By contrast, patients who do not have a better-seeing eye often take longer to reach the decision to stop treatment.

Very few studies have systematically reviewed outcomes of patients who discontinued intravitreal injection treatment for nAMD. One study showed that of patients who elected to discontinue injections, only 2.4% restarted treatment within 24 months; although some patients resumed treatment because of reactivation of the disease or declining visual acuity, e.g., in situations where there is a new retinal hemorrhage [21]. However, these situations are rare, and in our experience, most eyes remain free of symptoms, even if there is a recurrence of disease in eyes whose vision is already poor. In these cases, even if intravitreal anti-VEGF treatment is not resumed, patients should be followed more closely. After treatment is stopped, in most cases, the visual acuity in that eye remains stable [21]. Although there are no expert guidelines or formal criteria for when it is safe to stop treatment with anti-VEGF therapy, in the case of stable disease treated at long intervals between injections, some patients may elect to cease treatment or switch to a *pro re nata* (PRN) approach where treatment decisions are guided by OCT findings [3]. However, studies that have examined patients who met criteria to stop treatment by virtue of being disease-free for 48 weeks after receiving anti-VEGF injections every 12 to 16 weeks found that half of such patients resume treatment within six months to two years and do not always retain vision in the case of disease recurrence [10,22].

A multicenter retrospective study performed in Italy that reviewed the treatment records of 2302 individuals naïve to treatment who started anti-VEGF therapy for nAMD offers additional insights into the reasons why patients discontinue anti-VEGF therapy [23]. This study found that nearly 30% of the starting cohort discontinued treatment after at least two years of follow-up (655 individuals). In roughly half of those cases, the “clinical decision” to stop treatment was made based on a “poor response or non-response” to treatment. Another 3% of patients in this review of real-world, clinical data found that patients ceased therapy on their own for reasons unrelated to their response to treatment, but further details are lacking. While this study shows that it is not uncommon for patients to discontinue therapy in relation to an inadequate response to anti-VEGF therapy, the specific clinical data needed to gain insight into defining these outcomes which would allow the formulation of treatment recommendations, are generally lacking.

Although not a primary consideration, there are also both direct and indirect economic costs associated with intravitreal injection treatment. While compounded bevacizumab is relatively inexpensive, other anti-VEGF medications can be very costly [24]. The growing number of individuals with age-related eye diseases, including one out of every ten Americans above the age of eighty [25], and a limited number of eye care providers available to treat them [26], increases the desirability that patients and their providers elect to stop treatment when it is appropriate to do so. Studies have highlighted the importance of considering not only the cost of the drug, but also the physician’s time, extended clinic hours required to provide treatment, and any associated emergency visits [27].

When weighing the costs versus benefits of receiving intravitreal injections, the level of visual acuity in both the eye receiving treatment and the fellow eye must be considered. For example, in cases where a patient’s second eye has better vision, regardless of its treatment status, it tends to dominate vision-associated quality of life [28]. Small fluctuations in visual acuity in such an eye, which already has poor vision, have little impact on overall health-related quality of life. On the other hand, the costs of drugs and administration of intravitreal injections are generally favorable when considering the associated reduction in mortality risk and an improved health-related quality of life attributed to the incremental amount of visual acuity sustained by ongoing treatment with these agents [29]. However, these returns are proportional to the amount of vision conserved and decline over time. It is also important to note that the definition of low vision and visual disability from legal blindness is based on limitations in vision in the better-seeing eye [20].

Most patients who undergo treatment improve within the first year but return to baseline or worse within one to three years [30]. It is also well known that better baseline BCVA correlates with better long-term visual outcomes in patients with nAMD [31]. However, few studies have examined the long-term outcomes of patients (or eyes) that have reached very low levels of vision and have little vision left to preserve. One retrospective study of 1177 patients at a single center in the UK on a treat-and-extend protocol for nAMD sought to identify reasons for discontinuation of therapy [32]. The study reported that nearly half of patients failed to complete five years of follow-up: 251 patients stopped treatment because of death, 110 stopped treatment owing to stable disease, and 100 stopped treatment because it was deemed futile. In the group where further treatment was deemed futile, most patients stopped intravitreal injections of anti-VEGF medications because they had poor response to treatment at an average of four months. Not surprisingly, most of these patients also had permanent macular damage secondary to macular scarring or atrophy and had suffered significant vision loss (a median decline of 17 letters). It is also important to factor in that some patients stop treatment because of worsening general health, treatment dissatisfaction, or forgetting to return for scheduled appointments and becoming lost to follow-up [33,34]. Finally, patients may have other contributing components of the disease, such as macular scarring or geographic atrophy, or other retinal or optic nerve diseases, such as diabetic retinopathy or glaucoma. In some cases, these additional factors limit the utility of ongoing treatment. In fact, with the advent of treatments for GA in advanced dry AMD, many of the same principles regarding when to stop treatment similarly apply [34].

Receiving regular intravitreal injection of agents targeting VEGF decreases but does not prevent the recurrence of intraretinal fluid [35]. In cases where disease activity persists or recurs despite regular treatment, patients are often switched to another anti-VEGF medication to control disease activity or allow for extended intervals between injections to reduce the burdens of treatment [36,37]. While retina specialists strongly recommend ongoing treatment at specific intervals, attendance is often imperfect, and many patients become lost to follow-up. The most commonly reported barriers to treatment adherence include deterioration in health status, forgetting to attend or schedule a visit, and the discomfort of side effects from injection treatment itself [33,34]. Other studies have noted that lack of social support, difficulties with transportation, living far from the clinic, having lower levels of household income, identifying as African American, or being covered by Medicaid health insurance may contribute as impediments to accessing treatment of nAMD [38]. Age, visual acuity, and living far from their injection center have been shown to be risk factors for patients being lost to follow-up [34]. The role of non-shared language should also be considered [39], and interpreter services used to ensure clear, safe, and equitable communication between providers and patients.

Review of the clinical trial literature of anti-VEGF therapies provides few specific guidelines as to when it is appropriate to discontinue treatment or what defines end-stage nAMD. The Tenaya and Lucerne studies explicitly classified patients who stopped treatment; one of these categories contained patients who may have potentially stopped injections because of the lack of benefit, classified as “physician’s decision” as an endpoint [40]. Twelve patients stopped for this reason out of the 1329 enrolled across both studies. In contrast, MARINA and VIEW1 & VIEW2 studies do not disclose similar information about the reasons why patients discontinued treatment in these trials [4,41]. The authors propose that patients who demonstrate severe visual impairment (e.g., low vision category 2 as defined by the American Academy of Ophthalmology’s Vision Rehabilitation Preferred Practice Pattern^®^ recommendations for low vision [42]) at several successive visits should be considered functionally as having reached end-stage nAMD. This is equivalent to worse than 20/200 or as having “severe vision impairment” as established by the 2019 World Health Organization report on vision [43]. These patients typically have irreversible BCVA loss from subretinal fibrosis, macular scarring, or geographic atrophy [40]. Before stopping treatment, evaluation by a low vision specialist who can perform a careful low vision refraction should be completed. Such consultations may also provide better outcomes for patients by improving the visual acuity of the better-seeing eye [20], which is strongly linked to vision-related quality of life [28,44]. We hope that future clinical trials will specifically help set guidelines to define not only eligibility criteria for treatment but also help set the endpoint for these therapies. Translational research has also suggested that measuring VEGF levels may serve as a biomarker to guide decision-making in end-stage nAMD [45].

## 5. Conclusions

While intravitreal injections of anti-VEGF agents are effective at slowing the progression of nAMD, some patients inevitably reach the end stage of the disease. At this point, further injections will not stop progression or restore vision that has already been lost. Yet each procedure carries with it the same risks, costs, and burdens associated with the treatment at its outset. Building a strong patient–physician relationship is an essential part of personalized medicine in ophthalmology, necessary for guiding treatment and determining the point at which it should be discontinued. This relationship should be individualized to the needs of each patient and supplemented by the support of a low vision specialist who can help ensure that the best visual outcome is achieved for each case. In the future, a national or international consensus statement could also further support clinical practice by guiding treatment across the stages of nAMD, incorporating ophthalmological, medical, and social parameters.

## Data Availability

The original contributions presented in this study are included in the article. Further inquiries can be directed to the corresponding author.

## Abbreviations

The following abbreviations are used in this manuscript:

AMD	Age-related macular degeneration
nAMD	Neovascular age-related macular degeneration
VEGF	Vascular endothelial growth factor
CARE	CAse REport
CF	Counting fingers
RPE	Retinal pigment epithelium
BCVA	Best-corrected visual acuity
OCT	Optical coherence tomography
MARINA	Minimally classic/Occult trial of the Anti-VEGF antibody Ranibizumab in the treatment of neovascular age-related macular degeneration.
VIEW	Vascular Endothelial Growth Factor Trap-Eye: Investigation of Efficacy and Safety in Wet Age-Related Macular Degeneration
